# Location and home rules of cannabis use – Findings from marijuana use and environmental survey 2020, a nationally representative survey in the United States

**DOI:** 10.1016/j.pmedr.2023.102289

**Published:** 2023-06-23

**Authors:** Osika Tripathi, John Bellettiere, Sandy Liles, Yuyan Shi

**Affiliations:** a The Herbert Wertheim School of Public Health and Human Longevity Science, University of California San Diego, 9500 Gilman Drive, La Jolla, CA 92093, USA; b School of Public Health, San Diego State University, 5500 Campanile Drive, San Diego, CA 92182, USA

**Keywords:** Cannabis, Secondhand exposure, In-home smoking, Survey, Vaping, Dabbing, United States

## Abstract

Cannabis combustion and aerosolization may be associated with adverse health for users and nonusers through secondhand and thirdhand exposure. As cannabis regulation becomes more lenient, understanding where cannabis is used and whether homes have rules restricting use is needed. This study aimed to identify locations, presence of other people, and in-home rules of cannabis use in the United States (U.S.). This secondary analysis of 3,464 inhalation-based (smoking, vaping, dabbing) cannabis users in past 12 months drew from a cross-sectional probability-based online panel of 21,903 U.S. adults in early 2020, providing nationally representative estimates. We describe presence of other people and location at most recent use (smoking, vaping, dabbing, respectively). We also describe household restrictions on in-home cannabis smoking by cannabis smokers vs non-smokers, and by presence of children in home. Cannabis smoking, vaping, and dabbing most often occurred at users’ own homes (65.7%, 56.8%, and 46.9%, respectively). More than 60% of smoking, vaping, and dabbing occurred with someone else present. About 68% of inhalation-based cannabis users (70% and 55%, smokers and non-smokers, respectively) did not have complete restrictions on in-home cannabis smoking; among them, over a quarter lived with children under 18. In the U.S., inhalation-based cannabis use most commonly occurs at home, with others present and a substantial proportion of users lacking complete in-home cannabis smoking restrictions, raising risks of secondhand and thirdhand smoke exposure. These circumstances demand residential interventions for developing bans on indoor cannabis smoking, especially around vulnerable children.

## Highlights

• Cannabis smoking, vaping, and dabbing usually occur at home when others are present.
• Many inhalation-based cannabis users lack full restrictions on in-home smoking.
• Second & thirdhand cannabis smoke exposure is thus a potential public health concern.

## 1 Introduction

Cannabis is mainly used through inhalation-based modes: combustion (smoking) or aerosolization (vaping or dabbing).(Shi, 2021) Vaping involves using a device that heats up cannabis (natural or oils) to produce a mix of water vapor and cannabinoids, while dabbing involves flash-vaporization of cannabis concentrates (wax or shatter) at high temperatures. (Russell et al., 2018) Cannabis smoke contains carcinogens and toxicants, similar to cigarette smoke, some at higher concentrations in cannabis. Cannabis combustion has been associated with various respiratory outcomes. (Hall and Degenhardt, 2009) Health effects of aerosolization remain undetermined, but exploratory studies suggest that vaping and dabbing likely also lead to respiratory problems, though fewer compared to smoking. (Russell et al., 2018) Vaping may have other effects such as delayed impairment and normalization of use, while dabbing can result in acute risks such as excessive impairment and burn injuries. (Russell et al., 2018).

Analogous to public health concerns about tobacco smoking, cannabis smoking also generates harmful secondhand and thirdhand emissions, often involuntarily exposing nonusers. (Holitzki et al., 2017) Similar research on secondhand and thirdhand emissions from cannabis vaping and dabbing is limited. Children are especially susceptible due to their environmental interactions (hands frequently touching mouth and surroundings), rapidly developing physiology, and breathing more air per kilogram body weight than adults. (Sly and Flack, 2008) Emerging data indicate detrimental physical and mental health effects of secondhand and thirdhand cannabis emissions, especially for children, such as viral respiratory infections, cumulative adverse respiratory health outcomes, changes in gut biomes, and behavior problems. (Johnson et al., 2022, Kelley et al., 2021, Posis et al., 2019).

Cannabis use laws have become increasingly lenient in the United States (U.S.). As of September 2022, 37 states and Washington D.C. have legalized cannabis for medical use and 19 states and Washington D.C. have further legalized cannabis for adult use. (Marijuana Legality By State [WWW Document], n.d) Considering the potential harms associated with increased cannabis use and exposure to cannabis emissions, state and local governments have implemented policies to restrict cannabis use in public places. As of July 2022, 869 localities and 35 states/territories/commonwealths restrict cannabis use in some or all smokefree spaces, of which 468 localities and 21 states/territories/commonwealths prohibit cannabis smoking and vaping in some or all public places where tobacco is usually prohibited, such as workplaces, restaurants, bars, and gambling facilities. (American Nonsmokers’ Rights Foundation, n.d).

Despite potential harms of cannabis use and policy efforts to reduce cannabis emissions in public places, little is known about where cannabis is commonly used and whether homes have rules restricting cannabis use. The limited existing research in the U.S. and other countries on location of cannabis use and secondhand exposure has focused on cannabis smoking. (Anastasiou et al., 2020, Chu et al., 2019, Driezen et al., 2022, Schauer et al., 2020) No studies have examined locations of secondhand exposure to cannabis aerosolization (vaping or dabbing); in addition, virtually nothing is known about in-home rules or policies about cannabis smoking.

The objective of this study was to identify locations and presence of other people at the time of cannabis use in the U.S. Because different use modes are likely associated with different health consequences, we report data by inhalation-based mode: smoking, vaping, and dabbing. This study also aimed to characterize rules restricting cannabis smoking in home. Because children are particularly vulnerable to harms related to cannabis exposure, we assess home rules by the presence of children in the home.

## 2 Material and methods

### 2.1 Data source and study sample

This is a secondary analysis of data from the cross-sectional Marijuana Use and Environment Survey (MUES) 2020. MUES recruited 21,903 adult respondents (age 18+) between December 2019 and February 2020 from an address- and probability-based online panel maintained by the marketing research company Ipsos Public Affairs (formerly Gfk Group). The panel consists of approximately 55,000 panelists and is the largest probability-based online panel in the U.S, representing 97% of the U.S. adult population. Free internet services and/or devices were provided to panelists with limited access to internet. Survey weights are provided to make results representative of the U.S. census demographics: by gender, 51.9% were female; by race/ethnicity 63.8% were non-Hispanic White, 16.2% Hispanic, 11.6% non-Hispanic Black, 6.6% non-Hispanic other race, and 1.8% non-Hispanic multiple races; by education levels, 31.2% had a bachelors degree, 30.5% some college, 27.5% complete high school and 10.8% less than a high school education; with the mean age of 48.2 years (SD: 17.4). This panel has been frequently used in substance use studies estimating nationally representative statistics. (Azcarate et al., 2020, Cohn et al., 2015, Kennedy-Hendricks et al., 2016, Shi, 2021).

For our analyses, we restricted our data to respondents who reported using cannabis in the past 12 months by any of three inhalation-based modes: smoking, vaping, or dabbing. Of 21,903 respondents, 3,513 reported smoking, vaping and/or dabbing cannabis in the past 12 months. We further excluded 49 respondents for missing data on location or presence of other people at last inhalation-based use and on household rules about cannabis smoking inside of homes. In total, there were 3,464 inhalation-based cannabis users in the past 12 months with complete data. Of those, 3,162 reported smoking in the past 12 months, 1,644 reported vaping in the past 12 months, and 430 reported dabbing in the past 12 months.

This study does not constitute human subjects research that is subject to IRB review as it is a secondary analysis of the MUES data, which are de-identified.

### 2.2 Measures

Among inhalation-based cannabis users (smoked, vaped, and/or dabbed) in the past 12 months, the location of their last cannabis use for each mode was assessed as: 1) at own home, 2) at a friend’s or relative’s home, 3) in public places where tobacco smoking is not allowed (workplace, airport, bar/restaurant/club, children's playground or sports field, concert, hospital, movie theater, school, shopping mall, and sporting event), and 4) elsewhere (marijuana adult-use retail store, medical marijuana dispensary or cooperative, street, and other places not mentioned). For each mode, respondents were also asked whether they last used cannabis when someone else was around.

Rules for cannabis smoking inside homes were categorized as 1) complete restrictions (“no one is allowed anywhere”), and 2) incomplete or no restrictions (“allowed in some places”, “allowed everywhere”, or “did not make rules”). All respondents were also asked about children living in their household: children under 6, children 6–12, children 13–17. From these 3 variables on children, a variable indicating whether any children under age 18 lived in their home was created.

### 2.3 Statistical analysis

We computed the percentage and associated 95% confidence interval for the location and presence of other people at the last use for each of three modes: smoking, vaping, and dabbing. Because cannabis users could use more than one mode in the past 12 months, respondents could be in more than one of the three non-mutually exclusive categories.

We also computed the percentage and associated 95% confidence interval for the existence of complete restrictions on in-home cannabis smoking, by cannabis use status (smokers and non-smokers) and the presence of any children in home, among inhalation-based cannabis users.

All analyses were adjusted by survey sampling weights, making results generalizable to the U.S. adult population. Statistical software R was used for analyses.

## 3 Results

The prevalence of cannabis smoking, vaping, and dabbing in the past 12 months among the general population was 16.4%, 8.8%, and 2.6%, respectively. Among cannabis smokers in the past 12 months, 45.0% smoked cannabis one or more times per week, 16.5% smoked one or more times per month, 38.1% smoked less than once per month, and 0.4% had no data available. Among vapers in the past 12 months, 20.6% vaped one or more times a week, 16.8% vaped one or more times per month, 62.4% vaped less than once per month, and 0.4% had no data available. Among dabbers in the past 12 months, 17.5% dabbed one or more times a week, 24.6% dabbed one or more times per month, 57.8% dabbed less than once per month, and 0.8% had no data available.

Of 3,464 inhalation-based cannabis users: 9.8% reported smoking, vaping and dabbing in the past year, 50.1% reported only smoking, 8.4% reported only vaping, 0.2% reported only dabbing, 29.0% reported only smoking and vaping, 2.3% reported only smoking and dabbing, and 0.2% reported only vaping and dabbing. Among all inhalation-based cannabis users, 51.6% were male, 62.0% were non-Hispanic white, 14.9% non-Hispanic black, 15.5% were Hispanic, 4.5% were non-Hispanic other race/ethnicity, and 3.0% were non-Hispanic multiple races. The average age was 41.2 years (standard deviation: 15.2). Of all inhalation-based cannabis users, 34.3% lived in a state where medical and recreational cannabis use was legalized, 38.3% lived in a state where only medical cannabis use was legalized, 27.4% lived in a state where cannabis use was not legalized, and 1.7% did not have information available.

Table 1 reports the location and presence of other people at last cannabis use by mode. Smoking, vaping, and dabbing were most often reported at respondents’ own homes (65.7%, 56.8%, 46.9%, respectively), followed by friend’s/relative’s homes (23.0%, 22.2%, 35.7%, respectively). Use in public places was rare (2.7%, 7.8%, and 4.7% for smoking, vaping, and dabbing, respectively). At the time of last smoking, vaping, or dabbing, the proportion of instances with someone else around was 65.1%, 66.9%, and 75.8%, respectively. The presence of someone else most often occurred at users’ own homes (over 60% for each of the three modes), followed by friend’s/relative’s homes (over 35% for each mode).

**Table 1:** Estimated percentages for location and presence of other people at last cannabis use, by mode of use, among adult cannabis users in past 12 months – The 2020 Marijuana Use and Environment Survey.

	Last cannabis smoking	Last cannabis vaping	Last cannabis dabbing
	Cannabis smokers (n = 3,162)	Cannabis vapers (n = 1,644)	Cannabis dabbers (n = 430)
	% (95% CI)		
Location			
At own home	65.7 (64.3–67.4)	56.8 (54.6–59.0)	46.9 (42.8–51.1)
At a friend’s/relative's home	23.0 (21.6–24.4)	22.2 (20.4–24.1)	35.7 (31.8–39.7)
In public places where tobacco smoking is not allowed^1^	2.7 (2.2–3.3)	7.8 (6.7–9.1)	4.7 (3.2–6.8)
Elsewhere^2^	8.6 (7.7–9.5)	13.1 (11.7–14.7)	12.7 (10.2–15.8)

Was anyone around?			
Yes	65.1 (63.4–66.5)	66.9 (64.8–69.0)	75.8 (72.0–79.1)
At own home^3^	53.7 (51.7–55.7)	44.2 (41.5–47.0)	37.2 (32.7–41.9)
At a friend’s/relative's home^3^	32.6 (30.7–34.6)	31.0 (28.5–33.6)	45.6 (40.9–50.4)
In public places where tobacco smoking is not allowed^,^^1, 3^	3.7 (3.0–4.5)	9.6 (8.0–11.3)	4.6 (3.0–7.0)
Elsewhere 2, 3	10.0 (8.8–11.3)	15.2 (13.3–17.3)	12.6 (9.7–16.1)
No	34.9 (33.3–36.5)	33.1 (31.0–35.2)	24.2 (20.8–27.9)

1 Public places where tobacco smoking is not allowed included at workplace, at the airport, in a bar/restaurant/club, at a children's playground or sports field, at a concert, in a hospital, in a movie theater, in a school, at a shopping mall, and at a sporting event.

2 Elsewhere included in a marijuana adult-use retail store, in a medical marijuana dispensary or cooperative, on the street, and other places not mentioned.

3 Among those who used cannabis when someone else was around.

Table 2 reports the existence of in-home smoking rules for cannabis smoking among inhalation-based cannabis users. The proportion without a complete restriction on in-home cannabis smoking was 68.3% among inhalation-based cannabis users. Of the subset who smoked cannabis in the past 12 months, 69.6% did not have complete restrictions on in-home cannabis smoking. Of the subset who did not smoke cannabis in the past 12 months, 55.0% did not have complete restrictions on in-home cannabis smoking. Overall, among respondents without complete in-home cannabis smoking restrictions, 26.2% lived with at least one child under age 18.

**Table 2:** Estimated percentages, by age category of resident children, and by cannabis use status (smokers vs non-smokers), for in-home restrictions on cannabis smoking among adult inhalation-based cannabis users in the past 12 months – The 2020 Marijuana Use and Environment Survey.

	Inhalation-based Cannabis Users	Cannabis Smokers	Cannabis non-smokers
	(n = 3,464)	(n = 3,162)	(n = 302)
	% (95% CI)		
Complete restrictions on in-home cannabis smoking?			
Yes	31.7 (30.3–33.2)	30.4 (28.9–32.0)	45.0 (39.8–50.3)
No	68.3 (66.8–69.7)	69.6 (68.0–71.1)	55.0 (49.6–60.2)
Lived with child under 6^1^	13.3 (12.0–14.6)	13.1 (11.8–14.5)	15.2 (10.8–21.1)
Lived with child 6–12^1^	12.3 (11.1–13.6)	12.4 (11.2–13.8)	10.7 (7.0–15.9)
Lived with child 13–17^1^	9.3 (8.2–10.4)	9.7 (8.5–10.9)	4.3 (2.2–8.3)
Lived with at least one child under 18^1^	26.2 (24.6–27.9)	26.4 (25.0–28.2)	23.8 (18.2–30.4)

1 Among those who did not have complete restrictions on in-home cannabis smoking.

## 4 Discussion

While most studies focus on cannabis smoking behavior and exposure, our study was explicitly designed to understand *where* people commonly smoke cannabis and notably extends the literature by also examining *where* people commonly vape and dab cannabis. In this nationally representative U.S. adult sample who used cannabis through inhalation-based practices, cannabis smoking, vaping, and dabbing most often occurred at the respondent’s own home or at homes of people in their social network including friends or relatives. A public health concern highlighted by this study is that 68.3% of cannabis users did not have rules that completely ban cannabis smoking in their own home. Furthermore, among those without a complete home cannabis smoking ban, 26.2% lived with at least one child under 18. These circumstances raise concerns about cannabis emission secondhand and thirdhand exposure to children.

Our finding that cannabis smoking most commonly took place at users’ own homes and around others is consistent with existing studies. One U.S.-based observational study estimated that past-month cannabis smoke exposure in homes was ∼15 – 20%. (Driezen et al., 2022) Another study in New York City reported that two thirds of residents in subsidized housings were exposed to cannabis smoke in their home during the past year. (Anastasiou et al., 2020) Additionally, our analysis provides first evidence that more than 60% of the most recent smoking, vaping, and dabbing events occurred while someone else was present. This supports the commonly held view of cannabis as a socially experienced drug. Unfortunately, MUES did not identify the cannabis use status of the other people who were present. If they were using cannabis concurrently, secondhand emissions would be less concerning because they are already directly inhaling cannabis first-hand emissions. It should be noted, however, that multiple people smoking cannabis together likely generates additive effects on concurrent users themselves and puts all residents at greater risk of exposure to thirdhand emissions. Lastly, almost half (45%) of cannabis smokers in the past 12 months smoked one or more times per week, possibly indicating cumulative and higher risks of secondhand and thirdhand smoke exposure to children, other non-smoking residents, and anyone else around at the time of smoking.

Particularly relevant to children, this study found that among the nearly 70% of cannabis smokers lacking complete in-home cannabis smoking restrictions, over a quarter have children living in their home. While secondhand smoke dissipates from the environment within a few hours, thirdhand smoke has been shown to remain for up to 6 months.(Matt et al., 2017) Thirdhand tobacco smoke exposure has also been linked with adverse health effects in children, such as the changes in gut biomes observed for tobacco smokers and persons exposed to tobacco secondhand smoke, which may play a role in clinical outcomes in exposed children. (Kelley et al., 2021, p.) Secondhand and thirdhand exposure to cannabis emissions may be especially detrimental in early childhood as environmental exposures during this critical period may have a large impact on the child’s development and disease outcomes throughout life. (Landrigan and Goldman, 2011, Perlroth and Castelo Branco, 2017, Sly and Flack, 2008).

That a relatively higher proportion of public cannabis vaping (7.9%) than smoking (2.6%) was observed in our study has policy implications. Vaping is more discreet than smoking because it generates less odor and requires less time to use. There is concern that cannabis users may be more likely to vape than smoke in public places to avoid social stigma and circumvent smoke-free policies. While we applaud the wide adoption of laws that prohibit cannabis smoking and vaping in public places, such efforts may be insufficient to reduce exposure of nonusers, as cannabis is most often used at home, often without restrictions. Prevention and education programs are needed to promote complete restrictions on cannabis use at home, prioritizing homes with children. Laws prohibiting cannabis use in multi-unit residential properties should also be considered since shared walls and infrastructure lead to higher likelihood of shared emissions exposure.

This study has limitations. MUES only asked about the most recent location of cannabis use, which may not be the most often used location. As mentioned above, we also do not know the cannabis use status of other people present when the respondent was using cannabis. We hope future research can address this limitation, so we can move closer to estimating actual secondhand exposure. MUES did not directly measure secondhand exposure to cannabis, and when querying the location at which last use occurred for each mode, did not distinguish inside from outside-of-home use (such as on a patio or in a garden), which would be associated with different levels of exposure of those present. Future studies should ask about in-home vs. outdoor smoking. It is important to note that the majority of cannabis smokers in this study did not have a home cannabis smoking ban. So, while we do not know whether smokers’ last use was in the home, we know from decades of tobacco control research that instituting home smoking bans reduces exposure to children, and this is the first study to highlight a widespread lack of rules preventing cannabis smoking in the home. While we did not have survey questions about home rules concerning cannabis vaping, dabbing, or about tobacco and cannabis co-use, we hope future investigators will collect this important information. Co-use has been correlated with greater substance dependence and increased cancer risk among users, and secondhand and thirdhand exposure to mixed substances may also have unique health effects. (Cohn et al., 2021) Lastly, only 34.3% lived in a state where medical and recreational cannabis use was legalized, so responses on location of last use or report of use may be attenuated.

All information was self-reported and subject to social desirability and recall biases, which may result in under-reporting of cannabis use. The survey included one question about the presence of children at time of last use, but we do not report those data as we believe they may not reliably indicate exposure of children since the question was asked directly after questions on cannabis use. By design, our data regarding children living in the home were collected prior to any questions about cannabis use. Our data were collected a few months prior to the COVID-19 outbreak in the U.S. The ensuing response to the outbreak (quarantine, lockdown, social distancing, etc.) may have changed cannabis use behaviors, specifically frequency, location, and method of use, as a result of anxiety, boredom, or changes in access to the substance. (Boehnke et al., 2021, van Laar et al., 2020).

## 5 Conclusions

In the U.S., inhalation-based cannabis use most commonly takes place at users’ own homes when others are around, and a substantial proportion of cannabis users do not have complete restrictions on in-home smoking. These circumstances heighten the risk of exposure to the potential hazards of secondhand and thirdhand cannabis smoke—of special concern when cannabis users live with children, who are particularly vulnerable. Our findings impel public health practitioners and policy makers to develop interventions helping residents to create and enforce bans on in-home cannabis smoking. Also, to track in-home cannabis use trends and assess the impacts of prevention programs, we recommend improved measures of cannabis use locations and objective measures of exposure to cannabis emissions.

## Declaration of Competing Interest

The authors declare that they have no known competing financial interests or personal relationships that could have appeared to influence the work reported in this paper.

## Data Availability

The authors do not have permission to share data.
